# Targeted literature review exploring the predictive value of estimated glomerular filtration rate and left ventricular mass index as indicators of clinical events in Fabry disease

**DOI:** 10.1186/s13023-023-02936-7

**Published:** 2023-10-12

**Authors:** Ben Haycroft, Abigail Stevenson, Richard Stork, Stuart Gaffney, Philip Morgan, Karl Patterson, Ana Jovanovic

**Affiliations:** 1 Lumanity, Manchester, UK; 2 Chiesi Limited, Manchester, UK; 3 Northern Care Alliance NHS Foundation Trust, Salford, UK

**Keywords:** Fabry disease, eGFR, LVMI, Clinical events, Search, Studies, Patients

## Abstract

**Background:**

Fabry disease is a rare, progressive X-linked lysosomal storage disorder. It is caused by mutations in the *GLA* gene resulting in deficiency of α-galactosidase A (α-Gal A), leading to peripheral neuropathy, cardiovascular disease, stroke, end-stage renal disease, gastrointestinal disorders and premature death. Given the long-term nature of disease progression, trials in Fabry disease are often not powered to capture these clinical events. Clinical measures such as estimated glomerular filtration rate (eGFR) and left ventricular mass index (LVMI) are often captured instead. eGFR and LVMI are believed to be associated with long-term Fabry disease clinical events of interest, but the precise relationships are unclear.

**Objective:**

We aimed to identify published literature exploring the link between eGFR/LVMI and long-term clinical events in Fabry disease.

**Methods:**

A comprehensive literature search was conducted in Embase® and MEDLINE® (using Embase.com), and a targeted literature review was conducted. Studies reporting a quantitative relationship between eGFR and/or LVMI and clinical events in Fabry disease were extracted, and narrative synthesis was conducted to understand these predictive relationships.

**Results:**

Eight studies, consisting of seven patient-level retrospective analyses plus one prospective cohort study, met the inclusion criteria. Seven of these studies reported eGFR and six reported LVMI, with five reporting both. All studies presented results for either a composite measure including a range of key Fabry disease clinical events, or a composite outcome that included at least one key Fabry disease clinical event. All studies employed Cox proportional hazards survival modelling. The studies consistently reported that eGFR and LVMI are predictors of key clinical events in Fabry disease, with the findings remaining consistent regardless of the therapy received by patients in the studies.

**Conclusions:**

The evidence identified suggests that eGFR and LVMI outcomes may be appropriate indicators for long-term clinical events in Fabry disease, and all identified papers implied the same directional relationship. However, additional research is needed to further understand the specific details of these relationships and to quantify them.

## Background

Fabry disease, also known as Anderson–Fabry disease, is a rare, progressive X-linked lysosomal storage disorder. It is caused by mutations in the *GLA* gene resulting in deficiency of α-galactosidase A (α-Gal A), leading to the accumulation of globotriaosylceramide (GL-3 or Gb3) [1]. Accumulation of Gb3 in lysosomes may cause irreversible organ damage, resulting in serious long-term clinical manifestations such as cardiac events (cardiac failure, atrial fibrillation, ventricular arrhythmias, myocardial infarction), renal events (end-stage renal disease [ESRD], dialysis, renal transplant) and cerebrovascular events (stroke), and leading to death. [2, 3]

The severity, age of onset and progression of Fabry disease varies from person to person. Symptoms include Fabry crisis characterized by pain, fever and burning sensations, as well as gastrointestinal complications, headaches, impaired sweating, vertigo and hearing impairment.

Due to the long-term nature of clinical events in Fabry disease, clinical trials are often not designed and powered to capture these important clinical events, such as cardiac events, renal events, cerebrovascular events and survival. Outcomes such as estimated glomerular filtration rate (eGFR; a clinical indicator for renal outcomes) and left ventricular mass index (LVMI; a clinical indicator for cardiac outcomes) are often captured within clinical studies instead. Therefore, our study’s objective was to perform a targeted literature review (TLR) to identify and collate evidence for quantitative associations between eGFR and LVMI and long-term Fabry clinical events (mortality, cardiac complications, stroke, ESRD) that have a meaningful impact on survival, health-related quality of life and healthcare resource use [4]. Establishing the relationships between eGFR and LVMI and long-term clinical events in Fabry disease will allow for more valid and reliable modelling of long-term clinical outcomes of patients with Fabry disease, including within health economic models.

## Methods

### Search strategy

We conducted a comprehensive literature search in Embase® and MEDLINE® (using Embase.com). The TLR was restricted to studies published as full-text publications only, and searches were restricted to English language only but not restricted by date. The terms included in the search strategy for clinical indicators and relationships were informed by Fabry disease, eGFR, LVMI and clinical outcomes of interest.

Full details of the search strategy (search strings) are provided in Appendix 1.

Potentially relevant publications from the database search were reviewed and assessed to collate a final set of studies that attempted to quantify the relationship between eGFR and/or LVMI and clinical events (mortality, cardiac complications, stroke, ESRD). To determine the studies eligible for review, explicit inclusion and/or exclusion criteria were applied to the literature search results. Table 1 summarizes the key inclusion/exclusion criteria.

**Table 1:** Key criteria for study inclusion in the targeted literature review

	Inclusion criteria	Exclusion criteria
Population*	Fabry disease	Any other disease area
Outcomes	eGFR, or LVMI as indicators for clinical events (mortality, cardiac complications, stroke, ESRD)	Studies that do not report relationships for the outcomes of interest
Study design and publication types	Clinical studies reporting a relationship between eGFR/LVMI and clinical outcomes	Exclude comments, letters, editorials, news articles, case reports, in vitro studies, studies focused on animals, and articles reporting design of a study but not reporting results

Key: eGFR, estimated glomerular filtration rate; ESRD, end-stage renal disease; LVMI, left ventricular mass index

Notes: * The study was originally designed to search for studies in chronic kidney disease as well as Fabry disease, to provide some estimate of the relationship between eGFR and clinical events, in case of there being no results for Fabry disease. However, as Fabry disease studies exploring these relationships were identified, these studies were prioritized for data extraction and further review

### Screening

Primary (Level 1) screening was performed by a single reviewer, who reviewed each reference (title and abstract) identified in the literature search, applied basic study selection criteria (population and study design) and decided whether to include or exclude the study reference at this stage. Screening was followed by a 10% random quality control check by an independent reviewer.

For secondary (Level 2) screening, full-text articles were obtained for review. These were independently reviewed by a single reviewer against each eligibility criterion, followed by a 20% random quality control check by an independent reviewer. Data were extracted from the included papers after secondary screening.

## Results

### Search results

The electronic database searches were performed on 18 May 2021 and identified 5,236 records from Embase and MEDLINE. A total of five studies were removed as duplicates.

**Fig. 1:**
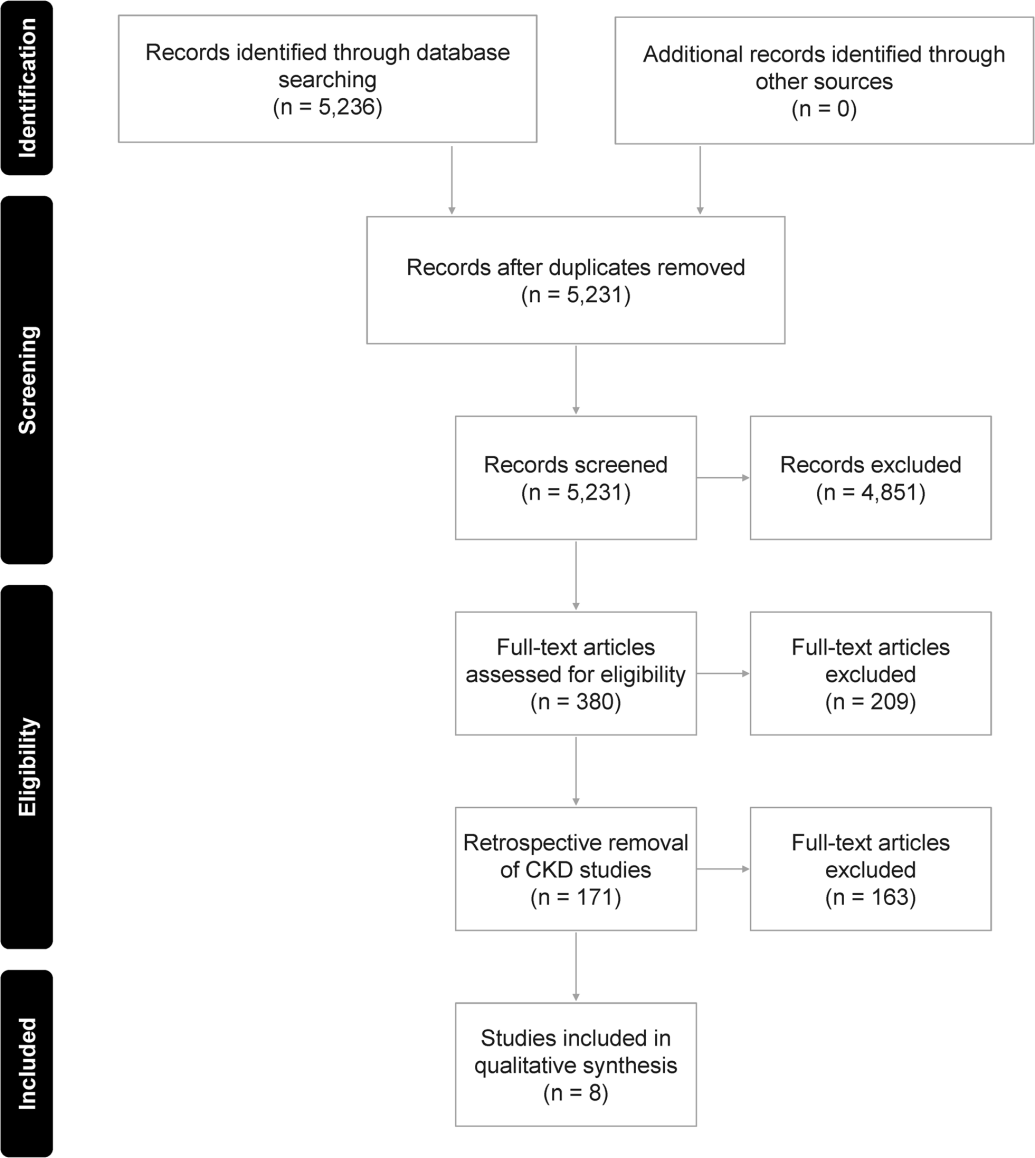
PRISMA diagram showing inclusion/exclusion of articles. Key: CKD, chronic kidney disease; PRISMA, Preferred Reporting Items for Systematic Reviews and Meta-Analyses. Note: CKD studies were included up to Level 2 screening

Following the screening of titles and abstracts, 4851 records were excluded, with 380 included for secondary screening. After secondary screening of full texts, eight articles were included for data extraction. Figure 1 presents the Preferred Reporting Items for Systematic Reviews and Meta-Analyses (PRISMA) flow diagram of studies included and excluded at different phases of the review. Papers were then screened as per the criteria in Table 1.

At the final stage of screening, a retrospective change to the protocol was made to increase the relevance of the TLR to Fabry disease.

### Overview of included studies

We identified eight studies of patients with Fabry disease that investigated the relationship between one or more clinical indicators (eGFR and/or LVMI) and Fabry clinical events after primary and secondary screening [5–12]. A summary of the key characteristics for these studies is shown in Table 2.

**Table 2:** Overview of included studies

Study	Location	Population	Measures reported	Clinical outcomes
Spinelli et al. [9]	Italy	Genetically proven FD with normal left ventricular ejection fraction	eGFR, LVMI	Cardiac events (defined as cardiac death, malignant ventricular tachycardia, atrial fibrillation and severe cardiac failure)
Graziani et al. [6, 15]	Italy	Genetically confirmed FD patients belonging to 20 different families	eGFR, LVMI	Composite measure of major events: cardiac failure, atrial fibrillation, stroke, progression to dialysis or eGFR decline
Hanneman et al. [8, 16]	Canada	Patients aged ≥ 18 years with gene-positive FD	LVMI	Cardiac failure, cardiac death
Feriozzi et al. [12, 18]	USA	Male and female patients aged ≥ 18 years with FD treated with agalsidase alfa	eGFR, LVMI	Myocardial infarction/cardiac failure, renal failure
Lenders et al. [10]	Germany	Classical or late-onset clinical phenotype form of FD including FD-typical signs and symptoms (patients with genetic variants of unknown significance and polymorphisms were not included)	eGFR	Myocardial infarction, ESRD
Siegenthaler et al. [13, 17]	Switzerland	Genetically confirmed FD patients	eGFR, LVMI	Composite measure including: cardiac failure, cerebrovascular events or death
Patel et al. [11]	Multiple	Previously untreated FD patients	eGFR	Mortality: cardiac events, stroke
Arends et al. [5, 14]	Netherlands, UK, and Germany	Adults with a definite FD diagnosis who were treatment-naïve and subsequently treated with either agalsidase alfa or agalsidase beta for ≥ 9 months	eGFR, LVMI	Clinical events: Renal event, cardiac arrest, cerebral events

Key: eGFR, estimated glomerular filtration rate; ESRD, end-stage renal disease; FD, Fabry disease; LVMI, left ventricular mass index

Note: * The study was originally designed to search for studies in chronic kidney disease as well as Fabry disease, to provide some estimate of the relationship between eGFR and clinical events, in case of there being no results for Fabry disease. However, as Fabry disease studies exploring these relationships were identified, these studies were prioritized for data extraction and further review

Five of these studies included both eGFR and LVMI as explanatory variables [5, 6, 9, 12, 13], two studies included only eGFR as a clinical indicator [10, 11], and one study reported results with LVMI as the only explanatory variable [8]. Five of the studies presented outcomes as part of a multi-functional composite measure that included key clinical outcomes of interest. [8–12]

The included papers reported a range of long-term clinical outcomes. Five of the studies presented results for composite outcomes for a single clinical function that included at least one of the clinical outcomes of interest—for example, a composite cardiovascular outcome that included myocardial infarction [8–12]. All five of these papers used composite endpoints that included results for relevant cardiac outcomes (such as myocardial infarction [9–12], cardiac death [8, 11] and cardiac failure [8, 9, 11, 12]), while two studies (Feriozzi et al. and Lenders et al.) also reported results for renal outcomes (including a worsening of CKD from Stage 1 to 3 [10] and renal failure [12]).

The other three studies all reported results for composite outcome measures that captured key clinical events across multiple functions: (1) cardiac failure, atrial fibrillation, stroke, CKD progression and eGFR decline [6]; (2) requirement of renal replacement, atrial fibrillation, pacemaker and/or implantable cardioverter defibrillator (ICD) implantation, cardiac failure, stroke and death [13]; and (3) worsening CKD and eGFR status; atrial fibrillation; admission for any rhythm disturbance; admission for congestive cardiac failure; implantation of an ICD or pacemaker; myocardial infarction; coronary artery bypass graft surgery or a percutaneous transluminal angioplasty intervention; stroke or transient ischaemic attack; and death. [5]

### Study/population characteristics

The key study and patient characteristics for the eight included studies are presented in Table 3. Out of the eight studies, seven were patient-level retrospective analyses [5–10, 12]. with only Siegenthaler et al. being a prospective cohort study. [13]

**Table 3:** Patient characteristics of included studies

Study	Treatment given	N	Mean age	Baselines		% Treatment-naïve	Follow-up (months)
				eGFR	LVMI		
Spinelli et al. [9]	N/A	96	Mean: 41.8 (SD ± 14.7)	Mean: 93.2 (SD ± 31.9)	Mean: 48.5 (SD ± 19.7)	17.6% naïve 4.2% with renal transplants 16.6% treated with ERT before baseline assessment 61.4% initiated ERT during follow-up	63 (IQR 37–85)
Graziani et al. [6, 15]	N/A	45	Mean: 52 (SD ± 16)	Mean: 87.6 (SD ± 31.1)	Mean: 135.2 (SD ± 78)	22.3% naïve 32 patients (71.1%) were on ERT, 24 (53.3%) with agalsidase alfa, and 7 (15.5%) with agalsidase beta One patient (2.2%) was receiving treatment with migalastat 6.6% previous kidney transplant	51.2 (SD ± 11.4)
Hanneman et al. [8, 16]	N/A	90	Mean: 44 (SD ± 15)	NR	Mean: 78 (SD ± 32)	22% on ERT at baseline 32% initiated after follow-up	43
Feriozzi et al. [12, 18]	Agalsidase alfa	560 (LVMI outcomes) 1093 (renal outcomes)	Age at agalsidase alfa initiation LVMI group: Mean 4.8 (SD ± 14.6) Renal group: Mean 44.1 (SD ± 14.8)	LVMI group: Mean 92.7 (SD ± 25.8) eGFR group: Mean 94.1 (SD ± 27.8)	LVMI group: Mean 54.6 (SD ± 20.4) eGFR group: Mean 54.1 (SD ± 20.5)	0%, all treated with agalsidase alfa	NR
Lenders et al. [10]	Agalsidase alfa or beta	54	Mean: 44.5 (SD ± 14.7)	Mean: 87.1 (SD ± 31.5)	NR	All patients naïve at baseline	71
Siegenthaler et al. [13, 17]	Agalsidase alfa or beta	104	Mean: 45 (SD ± 16)	NR	Median: 89 (IQR 64–127)	ACE inhibitor or ARB in 17 (16%) of patients at baseline, increasing to 34 (32%) at follow-up 48 (46%) patients were treated with agalsidase alfa and nine with agalsidase beta throughout the follow-up time	103 (range 59–155)
Patel et al. [11]	NR	2,869	Mean: 48.5 (SD ± 11.71)	Mean: 91 (SD ± 39.54)	NR	All patients naïve at baseline/data obtained before ERT initiated	NR
Arends et al. [5, 14]	Agalsidase alfa or beta	293	Median: 45 (range: 18–79)	Median: 91 (range: 5–140)	Median: 51 (range: 16–149)	All patients naïve at baseline	82 (range 9.6–185)

Key: ACE, angiotensin-converting enzyme; ARB, angiotensin receptor blocker; eGFR, estimated glomerular filtration rate; ERT, enzyme replacement therapy; FD, Fabry disease; IQR, interquartile range; LVMI, left ventricular mass index; N, number; NA, not applicable; NR, not reported; SD, standard deviation

Patient characteristics were largely similar in the included studies. The age range was 41–52 years, with an average age of 45. All papers except Hanneman et al. and Siegenthaler et al. had a sex composition within ± 10% of having an even male/female distribution. For all papers that reported mean eGFR [5, 6, 9–13], the spread of mean baseline values was narrow (87–‍93 mL/min/1.73 m^2^). For LVMI, four papers used unadjusted LVMI (g/m^2^) [14–17] and two adjusted for height (g/m^2.7^). [18, 19]. Siegenthaler et al. used the notation LVMMI when reporting results. Given this is a synonym of LVMI, LVMI is used throughout when discussing this paper, for simplicity. The range in LVMI for these papers was 52 g/m^2^–171.6 g/m^2^ for males and 46 g/m^2^–89.8 g/m^2^ for females. For papers reporting height adjusted LVMI this was 57.8 g/m^2.7^–109 g/m^2.7^ and 42.3 g/m^2.7^–75 g/m^2.7^, for males and females respectively.

The proportion of treatment-naïve patients at baseline varied among the studies. Three included patients who had undergone enzyme replacement therapy (ERT) before baseline assessment [6, 8, 9]; ERT usage in these studies ranged from 17% [9] to 71% [6]. Three of the studies included patients who initiated ERT during the follow-up period_,_ ranging from 32% [8] to 61% [9] of patients. Three studies included patients that were all treatment-naïve at baseline and did not undergo any treatment for the entirety of the follow-up period [5, 10, 11]. Spinelli et al., Graziani et al. and Siegenthaler et al. were the only studies to include patients who had undergone renal transplantation, with transplantation rates of 4.2%, 6.6% and 8%, respectively. [6, 9, 13] Siegenthaler et al. was the only study to explicitly state any co-medications being given to patients alongside treatments indicated for Fabry disease, either at baseline or during follow-up. In this paper, patients received either an angiotensin-converting enzyme (ACE) inhibitor or an angiotensin receptor blocker (ARB) (16% of patients at baseline, increasing to 32% at follow-up) [13]. Feriozzi et al. was the only study to include patients who had all initiated ERT before baseline assessment; all patients received agalsidase alfa. [12]

Six of the included studies reported length of follow-up [5, 6, 8–10, 13]. The average length of follow-up was 69 months, with a range of 43–103 months. [8, 13]

### Relationship between eGFR and LVMI on clinical outcomes in Fabry disease

Table 4 presents the relationships for the prognostic impact of baseline eGFR/LVMI levels on experiencing a relevant clinical outcome during response to treatment.

**Table 4:** Study predictive relationships reported

Study	Method of analysis	Results	
		OR	HR
Spinelli et al. [9]	Univariate Cox analysis with predictor variable thresholds established by ROC curve analysis	NR	Predictors of events*: LVMI (g/h^2.7^) > 54.3: 1.022 (1.008–1.036), p = 0.002 eGFR ≤ 69: 0.978 (0.961–0.996), *p* = 0.016
Graziani et al. [6, 15]	Univariate and multivariate Cox regressions	NR	Predictors of events*: eGFR: 0.97 (0.95–0.99), *p* 0.008 LVMI (g/m^2^) (univariate): 1.01 (1.00–1.01) *p* < 0.001 LVMI (g/m^2^) (multivariate): 1.01 (1.00–1.03) *p* = 0.03
Hanneman et al. [8, 16]	Univariate and multivariate Cox regressions	NR	**HR for 5 g/m**^**2**^** increase in LVMI** 1.1 (1.1–1.2), *p* < 0.001 (univariate) 1.1 (1.04–1.2), *p* < 0.001 (multivariate)
Feriozzi et al. [12, 18]	Multivariate Cox regression analysis	NR	**HR for cardiovascular event** LVMI (g/h^2.7^) at baseline (abnormal vs normal*) 1.57 (1.21–2.05) *p* < 0.001 eGFR at baseline (abnormal vs normal) 1.33 (1.04–1.70) *p* = 0.021 **HR for renal event** LVMI (g/h^2.7^) at baseline (abnormal* vs normal) 1.90 (0.94–3.85) *p* = 0.074 eGFR at baseline (abnormal* vs normal) 5.88 (2.73–12.68) *p* < 0.001 * Abnormal LVMI defined as > 50 g/h^2.7^ in males and > 48 g/m^2.7^ in females; abnormal eGFR defined as < 90 mL/min/1.73 m^2^
Lenders et al. [10]	Cox proportional hazards model	NR	Patients without endpoints had an average eGFR at baseline of 102.6 (± 26.6) compared to 71.3 (± 28.1) HR for endpoint-free survival of on ERT patients with an eGFR = < 75 mL/min/1.73 m^2^**:** 4.77 (1.93–11.81), *p* < 0.001 HR for cardiovascular endpoints after ERT initiation endpoint-free survival for patients with an eGFR ≤ 75 mL/min/1.73 m^2^: 3.59 (1.15–11.18); *p* = 0.03
Siegenthaler et al. [13, 17]^†^	Univariate and multivariate Cox regressions	NR	HR for occurrence of primary endpoint per SD increase in LVMMI: Crude (2.14, CI 1.52–3.01, *p* < 0.001), Model 1 (1.65, CI 1.06–2.58, *p* = 0.03), Model 2 (1.67, CI 1.04–2.73, *p* = 0.03), Model 3 (1.67, CI 1.04–2.73, *p* = 0.03) HR for occurrence of primary endpoint per SD increase in eGFR: Crude (0.42, CI 0.28–0.61, *p* < 0.001), Model 1 (0.45, CI 0.27–0.74, *p* = 0.002), Model 2 (0.42, CI 0.24–0.74, *p* = 0.003), Model 3 (0.45, CI 0.25–0.83, *p* = 0.01) HR for occurrence of primary endpoint for patients with eGFR < 90 mL/min/1.73 m^2^ at baseline, Crude (6.38 CI 1.91–21.33 *p* = 0.003) Model 1 (4.3 CI 1.06–17.52 *p* = 0.04) Model 2 (4.88 CI 1.12–21.24) Model 3 (4.41 CI 0.97–20.06) HR for occurrence of primary endpoint for patients with LVMMI > 107 g/m^2^ at baseline, Crude (4.28, CI 1.58–11.56 *p* = 0.004), Model 1 (1.79, CI 0.55–5.82 *p* = 0.37), Model 2 (1.83, CI 0.56–5.97 *p* = 0.32), Model 3 (1.75 CI 0.54–5.69 *p* = 0.35) Higher LVMMI and lower eGFR at baseline associated with a greater risk of developing an adverse clinical event (HR 2.21 [1.43–3.42], *p* < 0.001 for LVMMI, HR 0.41 [0.27–0.62], *p* < 0.001 for eGFR) (per SD increase in LVMMI and SD decrease in eGFR)—Excluding atrial fibrillation Crude model contained only LVMMI/eGFR as predictor, Model 1 included age and gender, Model 2: Model 1 + transplant status, dialysis status and Model 3: Model 2 + hypertension status and baseline LVMMI/eGFR
Patel et al. [11]	Univariate logistic regression models, eGFR not considered in multivariate model as not selected during stepwise selection	OR of experiencing a CV event for patients with low eGFR (< 60 mL/min/1.73 m^2^**)** Men OR: 2.33 (1.22–4.45), *p* < 0.05 Women OR: 3.85 (1.78–8.32), *p* < 0.0001	NR
Arends et al. [5, 14]	Univariate and multivariate Cox regressions	NR	HRs of the additional potential prognostic variables on the clinical event rate, adjusted for age at start of ERT, sex and phenotype Mixed-effect models **eGFR** (per −10 mL/min/1.73 m^2^) HR: 1.19 (1.11–1.27), p < 0.001 **eGFR** < 60 mL/min/1.73 m^2^ HR: 3.58 (2.21–6.05), p < 0.001 **LVMI** (per 10 g/m^2.7^) HR: 1.25 (1.08–1.45), *p* < 0.01 Multivariate **eGFR** (per −10 mL/min/1.73 m^2^) HR: 1.12 (1.03–1.22), *p* < 0.01 **LVMI** (per 10 g/m^2.7^) HR: 1.16 (0.99–1.36), *p* > 0.05 Multivariate analysis with eGFR as dichotomous variable **eGFR** (< 60 mL/min/1.73 m^2^) 2.66, CI 1.45–4.90, *p* = 0.002 **LVMI** (per 10 g/m^2.7^) 1.19, CI 1.02–1.38, *p* = 0.028 Multivariate analysis, excluding renal events **eGFR** (per -10 mL/min/1.73 m^2^) 1.01, CI 0.93–1.10, *p* = 0.78 **LVMI** (per 10 g/m^2.7^) 1.19, CI 1.02–1.38, *p* = 0.03

Key: CI, confidence interval; CV, cardiovascular; eGFR, estimated glomerular filtration rate; ERT, enzyme replacement therapy; HR, hazard ratio; LVMI, left ventricular mass index; LVMMI, left ventricular myocardial mass index; NR, not reported; OR, odds ratio; ROC, receiver operating characteristic; SD, standard deviation

*All events described in Table 2

^†^‘increase’ is based on this papers’ authors interpretation of the study results and conclusions, rather than the author of said paper’s results table

To estimate hazard ratios (HRs) between eGFR and/or LVMI and clinical outcomes of interest, all the identified papers employed Cox proportional hazards survival modelling. This modelling approach estimates the impact that variables have on the risk of a specific event happening at any point in time. The HRs obtained from this analysis allow for an interpretation of the impact that variables have on survival, with an HR < 1 implying decreased risk of an event and an HR > 1 implying an increased risk.

### Estimated glomerular filtration rate

Seven of the eight included papers presented results for eGFR, and generally showed a negative relationship between low/decreasing eGFR and clinical events [5, 6, 9–13]. Six of these studies presented results using an HR based on baseline eGFR, with Patel et al. providing odds ratios (ORs) based on baseline eGFR.

Spinelli et al. applied receiver operating characteristic (ROC) curve analysis to identify the threshold level that provided the best cut-off for outcome prediction for eGFR, resulting in a threshold of 69 mL/min/1.73 m^2^. Cox analysis showed an HR of 0.98 (*p* = 0.016) for cardiac events for eGFR (mL/min/1.73 m^2^). This was similar to the Cox regression results from Graziani et al. (HR 0.97; *p* = 0.03 for major clinical events). Feriozzi et al. undertook separate analyses for cardiovascular and renal events depending on whether a patient’s eGFR was deemed abnormal or normal (abnormal defined as eGFR < 90 mL/min/1.73 m^2^). The HRs for patients with abnormal eGFRs were reported as 1.57 (*p* = 0.021) for cardiovascular events and 5.88 for renal events (*p* < 0.001). Siegenthaler et al. explored the impact of baseline eGFR on a composite outcome of requiring renal replacement therapy (kidney transplantation or chronic dialysis), newly diagnosed atrial fibrillation of any type (paroxysmal/persistent), pacemaker and/or ICD implantation, hospitalization due to decompensated cardiac failure, cerebrovascular events (stroke or transient ischaemic attack) and death, whichever occurred first. The authors employed four models to estimate the prognostic effect of eGFR < 90 mL/min/1.73 m^2^. The crude model contained only LVMI/eGFR as a predictor; Model 1 included age and gender; Model 2 included Model 1 plus transplant status and dialysis status; and Model 3 included Model 2 plus hypertension status and baseline LVMI/eGFR. The crude model gave the highest HR of 6.38 (*p* = 0.003), while Models 1–3 gave consistent HRs of 4.3–4.41 for eGFR < 90 mL/min/1.73 m^2^. This paper also reported an HR of 0.42 (*p* < 0.001) per standard deviation (SD) increase in eGFR.

Lenders et al. explored outcomes for treatment-naïve patients receiving ERT based on baseline eGFR values against risk of myocardial infarction and progression to ESRD. Fifty percent of patients who initiated ERT experienced new clinical endpoints. Of these patients, the average eGFR at baseline was 71.3 (± 28.1), compared with 102.6 (± 26.6) for those who had no new recorded endpoints. After ERT initiation, patients with an eGFR of ≤ 75 mL/min/1.73 m^2^ faced an HR of 4.77 (*p* < 0.01) for endpoint-free survival and 3.59 (*p* = 0.03) for cardiovascular endpoints. [10]

Patel et al. was the only study to report ORs. This study aimed to estimate ORs of experiencing cardiac events (cardiac failure, myocardial infarction, cardiac-related death) for patients with eGFR < 60 mL/min/1.73 m^2^, stratified by sex. For men, the odds of experiencing an event with eGFR < 60 mL/min/1.73 m^2^ versus those with eGFR > 60 mL/min/1.73 m^2^ were 2.33 (*p* < 0.05). For women, the odds were 3.85 (*p* < 0.0001).

Finally, Arends et al. presented results for eGFR as a dichotomous variable (above or below 60 mL/min/1.73 m^2^) and as a continuous variable (per −10 mL/min/1.73 m^2^). Mixed-effects and multivariate analyses were used to estimate the relationship. Results showed a consistent but varying impact of eGFR on outcomes. Mixed-effects modelling gave an HR of 1.19 (*p* < 0.001) for a 10 mL/min/1.73 m^2^ decrease in eGFR and 3.58 (*p* < 0.001) for eGFR below 60 mL/min/1.73 m^2^. For multivariate analysis, the HR was 1.12 (*p* = 0.002) for a decrease in eGFR. When used as a dichotomous variable, eGFR gave an HR of 2.66 (*p* = 0.002). When renal events were excluded from the outcome measure, the HR dropped to 1.01 and the result became statistically insignificant (*p* = 0.78).

### Left ventricular mass index

Six of the eight studies contained LVMI as an explanatory variable of clinical outcomes [5, 6, 8, 9, 12, 13]. All six showed LVMI to be a predictor of clinical outcomes of interest in univariate and/or multivariate analysis. Two of the studies calculated LVMI by correcting to height powered to 2.7 [18, 19] and four calculated LVMI by normalizing to body surface area. The former method to calculate LVMI changes the upper limit for normal LVMI; as such, the following papers are discussed separately by LVMI calculation method [20].

For the papers that calculated LVMI using body surface area, Siegenthaler et al. and Graziani et al. estimated the relationship based on baseline values of LVMI. Graziani et al. undertook both multivariate and univariate analysis of LVMI on patients experiencing one of the following cardiac events after extensive baseline evaluation: new-onset atrial fibrillation, sustained ventricular arrhythmias, cardiac failure, or pacemaker/ICD implantation. The authors found an HR of 1.01 in both the univariate and multivariate analyses. The analysis showed it was a significant predictor, but the level/change of LVMI associated with this increased level of risk was not reported. Siegenthaler et al. employed the same three models as discussed above for eGFR (Models 1–3) but using LVMI as the dependent variable. For patients with a left ventricular myocardial mass index of > 107 g/m^2^ at baseline, the crude model gave an HR of 4.28, and Models 1–3 all gave similar HRs of 1.75–1.83. The paper also reported HRs of 1.65–2.14 for all models per SD increase in LVMI. Hanneman et al. and Arends et al. both analysed the impact on a composite outcome of clinical events. For the former, this was cardiac events (ventricular tachycardia, severe cardiac failure or cardiac death), and for the latter this was renal events, cardiac events, cerebral events or death due to a change in LVMI. Hanneman et al. evaluated hazards based on a 5 g/m^2^ increase in LVMI, while Arends et al. evaluated hazards based on a 10 g/m^2^ increase. Both papers conducted univariate and multivariate analyses and gave HRs of similar magnitudes (Hanneman et al.: 1.1 [*p* < 0.001]; Arends et al.: 1.16 [*p* > 0.5]–1.25 [*p* < 0.01]). Interestingly, in the Arends et al. study, whether or not LVMI significantly predicted outcomes was dependent on whether eGFR was included as either a continuous or dichotomous variable.

For the two papers that calculated LVMI adjusting for height powered to 2.7, Feriozzi et al. presented multivariate regression results for abnormal versus normal baseline LVMI on either cardiovascular or renal events (abnormal LVMI was defined as > 50 g/h^2.7^ in male and > 48 g/h^2.7^ in female patients). The HR for a patient with abnormal LVMI experiencing a myocardial infarction/cardiac failure was 1.57 (*p* < 0.001) and for renal failure was 1.9 (*p* = 0.074) versus those with normal LVMI. Spinelli et al. used ROC curve analysis to obtain the LVMI threshold that best predicts cardiac events defined as one of the following: cardiac death, malignant ventricular tachycardia, atrial fibrillation and severe cardiac failure development. This study demonstrated that this LVMI threshold was 54.3 g/h^2.7^, with regression analysis demonstrating a modest HR of 1.022 (*p* < 0.002) per LVMI change (g/h^2.7^).

## Discussion

Our review identified eight published studies that attempted to evaluate a quantitative relationship between eGFR and/or LVMI and key clinical events in Fabry disease, including mortality, cardiac complications, stroke and ESRD. Seven of the included studies were patient-level retrospective analyses [5, 6, 8–12], while one [13] was a prospective cohort study. The included studies demonstrated heterogeneity in patient treatment backgrounds, including the following: treatment-naïve at baseline assessment or during follow-up [5, 10, 11], having had ERT at baseline assessment [6, 8, 9, 12], initiating ERT during the follow-up period [8, 9, 13], renal transplant [6, 9], and ACE inhibitor/ARB [13]. Seven of the included studies contained eGFR as an explanatory variable, and six contained LVMI. Reported outcomes also varied across studies, with some opting for a composite measure [5, 6, 11, 13] and some opting for an isolated or range of isolated outcome(s) [8–10, 12]. Patient populations in the included studies were broadly similar, except for some variability in LVMI; however, exclusion of the study by Graziani et al. considerably reduces this variation.

There was a clear consensus among the included studies that the LVMI measurement in isolation has a key influence on patients experiencing Fabry long-term clinical events, with the results of these studies identifying links for both composite events and cardiac events. This held true for studies whether they used continuous or dichotomous definitions for LVMI as well as for studies that estimated LVMI using surface area or correcting for height powered to 2.7. The relationship also held across studies that had varying compositions of treatments received. However, there was reported uncertainty on whether the predictive value of LVMI is outweighed by the contribution of eGFR. Further research is needed to understand the contribution of eGFR when trying to isolate the prognostic power of LVMI for different Fabry disease phenotypes. [5]

The evidence for using eGFR to model a predictive relationship was also largely supported by the literature identified in this review. This relationship was consistent across the different types of analysis reported, such as assessment of eGFR as a continuous variable or differences between defined groups.

Arends et al. was considered to be the best account for predictive relationships between eGFR/LVMI and Fabry clinical events, as this study employed both mixed-effects and multivariate analysis and the use of both continuous and dichotomous modelling of LVMI and eGFR. LVMI remained a significant predictor of clinical events in all modelling methods except when eGFR was included as a dichotomous variable, implying the need to include eGFR with LVMI in models with an appropriate cut-off value. eGFR was a significant predictor of outcomes in both mixed-effects and multivariate analyses. eGFR was not a significant predictor when renal events were excluded from the clinical outcome, suggesting that eGFR and LVMI may independently contribute to clinical outcomes of their respective functions.

It is worth considering the findings of this review alongside other published literature reviews that have aimed to identify predictive relationships for alternative clinical indicators commonly collected in Fabry disease trials. Cha et al. undertook a TLR to assess the role of the Mainz Severity Score Index (MSSI) as a prognostic tool for patients with Fabry disease. The included papers generally showed a significant association between MSSI and key Fabry outcomes. However, similar to our TLR, a range of composite outcomes were assessed, making it difficult to determine the predictive power of MSSI. Further studies would need to be undertaken to quantify this [21]. Another literature review carried out by Azimpour et al. aimed to assess the relationship between levels of Gb3 and lyso-Gb3 and key clinical events in patients with Fabry disease, with this TLR again showing an association between Gb3 and lyso-Gb3 and cardiac, renal and cerebral events. [22]

Our review identified a number of highly relevant studies that estimated a relationship between key clinical outcomes in Fabry disease and eGFR and/or LVMI. It highlighted that the majority of studies drew similar conclusions, with a consistent trend observed between eGFR/LVMI and the outcomes of interest, and that this did not vary when different methodologies were adopted to measure the quantitative relationship (univariate, multivariate and mixed-effects).

A limitation of this review was that few papers included the key driver variables in forms other than continuous, and all studies reported only the impact of a patient’s baseline eGFR/LVMI. Only Arends et al. investigated both continuous and dichotomous measures for eGFR, and the results from this analysis were less conclusive than those for other methods. Any interpretation of these findings should be considered with the caveat that these relationships are from baseline and across patients, rather than measuring the impact of changes for each individual patient over time. Also, univariate analysis was the most common form of regression analysis, which unfortunately does not aid the understanding of the impact of other variables that could also be driving the relationship.

On a similar note, we did not set out to investigate the relationship between eGFR and LVMI themselves. The clinical events reported in the studies included in this review were all considered as part of a composite outcome. As such, the conclusions of this review are limited to the impact of eGFR and LVMI on clinical events when considered in general and within specific functions, rather than the impact on individual events. Mean eGFR reported across the papers was 87–93 mL/min/1.73 m^2^, which falls at the lower end of what is considered normal (90–120 mL/min/1.73 m^2^). This could therefore reduce the feasibility of using eGFR to predict future events in patients with worse renal function decline. Furthermore, the length of follow-up in some of the papers may not be long enough to capture the long-term clinical events of interest and could therefore understate the predictive power of eGFR/LVMI due to too few events being captured in the follow-up period. This could be especially true for females, in which disease progression is slower [23]. Lastly, the included studies demonstrated heterogeneous patient populations, which did not allow for the quantitative relationship between eGFR/LVMI and long-term clinical events in different Fabry disease phenotypes to be explored.

## Conclusions

In conclusion, quantitative measures of the relationship between eGFR and/or LVMI and clinical events were identified across multiple publications in this review. The results presented could be used to inform a predictive relationship between eGFR and LVMI and clinical events, with the study from Arends et al. potentially providing the most robust results. While the direction of the relationship was unanimous among the included studies, its magnitude was not. It is also not entirely clear from the included studies at what level eGFR and LVMI are predictive of events, nor whether it is better to use a cut-off point or increments.

The identified evidence supports a relationship between eGFR and/or LVMI and clinical events, but questions remain around which clinical events are best predicted by eGFR or LVMI and the relationship and contribution of eGFR and LVMI in each of these predictions.

## Data Availability

All data included in the targeted literature review was from published journal articles. The data extraction worksheets are available on request from AS or the Chiesi GRD HEOR team.

## Appendix 1: Search strategy for searching in Embase.com (conducted 18 May 2021)

**Table:**

Number	String	Hits
1	'estimated glomerular filtration rate'/syn OR 'estimated glomerular filtration rate'/exp OR 'egfr':ab,ti,kw OR 'glomerular filtration rate':ab,ti,kw OR 'glomerular filtration':ab,ti,kw OR 'glomerulus filtration rate'/syn OR 'glomerulus filtration rate'/exp OR gfr:ab,ti,kw	230,673
2	'heart left ventricle mass'/syn OR 'heart left ventricle mass'/exp OR 'left heart ventricle mass':ab,ti,kw OR 'left ventricular mass':ab,ti,kw OR 'left ventricular mass index'/syn OR 'left ventricular mass index'/exp OR 'left ventricular mass index':ab,ti,kw OR 'left ventricular hypertrophy'/syn OR 'left ventricular hypertrophy'/exp OR 'left ventricular hypertrophy':ab,ti,kw OR lvmi:ab,ti,kw OR 'heart left ventricle hypertrophy'/exp	61,192
3	#1 OR #2	288,768
4	'fabry disease'/exp	8,042
5	(((galactosidase OR gla OR anderson*) NEAR/3 (disease OR deficiency OR syndrome)):ab,ti,kw) OR ((angiokeratoma NEAR/3 diffus*):ab,ti,kw) OR 'ceramide trihexosidosis':ab,ti,kw OR fabry*:ab,ti,kw OR glycosphingolipidosis:ab,ti,kw OR 'dystopic lipidosis':ab,ti,kw OR 'mckusick 30,150':ab,ti,kw OR 'ruiter-pompen':ab,ti,kw OR 'ruiter pompen':ab,ti,kw OR 'sweeley-klionsky':ab,ti,kw OR 'sweeley klionsky':ab,ti,kw OR 301500:ab,ti,kw	9,627
6	#4 OR #5	11,134
7	'chronic kidney failure'/exp OR 'end stage renal disease'/exp OR 'ckd':ab,ti,kw OR 'esrd':ab,ti,kw	220,199
8	'correlation analysis'/exp OR 'correlational study'/exp OR 'predictor variable'/exp OR 'predictor*':ab,ti OR 'predictors'/exp OR 'disease marker'/exp OR 'disease marker*':ab,ti OR 'proxy'/exp OR proxy*:ab,ti OR 'biological marker'/exp OR 'biological marker*':ab,ti OR 'intermediate*':ab,ti OR 'marker'/exp OR marker*:ab,ti OR 'regression analysis'/exp OR regression*:ab,ti OR 'meta regression analysis'/exp OR ((meta NEAR/2 regression):ab,ti) OR 'multivariate analysis'/exp OR multivaria*:ab,ti OR (((relation* OR associat* OR correlat* OR link*) NEAR/4 (outcome* OR variabl* OR endpoint* OR 'end-point*' OR indicator* OR marker*)):ab,ti) OR 'validation study'/exp OR surroga*:ab,ti OR causal:ab,ti OR predict*:ab,ti,kw	6,153,813
9	#3 AND #6 AND #8	399
10	#3 AND #7 AND #8	24,744
11	#9 OR #10	25,070
12	'case study':it OR 'case report':it OR 'abstract report':it OR editorial:it OR letter:it OR comment:it OR note:it OR 'case report'/exp OR 'case study'/exp OR 'editorial'/exp	5,187,318
13	'animal'/exp NOT ('animal'/exp AND 'human'/exp)	5,606,712
14	(review:it OR 'literature review':it) NOT ('meta-analysis':it OR 'meta-analysis as topic'/mj OR 'systematic review':ti OR 'systematic literature review':ti OR 'meta-analysis':ab,ti OR 'meta analysis':ab,ti)	2,639,376
15	#12 OR #13 OR #14	13,135,018
16	#11 NOT #15	22,413
17	#16 AND ([conference abstract]/lim OR [conference paper]/lim OR [conference review]/lim OR [data papers]/lim OR [editorial]/lim OR [letter]/lim OR [note]/lim OR [short survey]/lim)	10,302
18	#16 NOT #17	12,111
19	'heart disease'/exp OR ((cardiac NEAR/2 complication):ab,ti,kw)	2,093,268
20	'mortality'/exp OR 'mortality rate'/exp OR 'survival'/exp OR 'survival time'/exp OR 'survival analysis'/exp	2,143,543
21	'mortality':ab,ti	1,202,217
22	'cerebrovascular accident'/exp OR (((asymptomatic OR silent) NEAR/2 stroke):ab,ti,kw)	347,796
23	#19 OR #20 OR #21 OR #22	4,388,527
24	#18 AND #23	5,236

## Abbreviations

ACE: Angiotensin-converting enzyme
α-Gal A: Alpha-galactosidase
ARB: Angiotensin receptor blocker
CI: Confidence interval
CKD: Chronic kidney disease
CV: Cardiovascular
eGFR: Estimated glomerular filtration rate
ERT: Enzyme replacement therapy
ESRD: End-stage renal disease
Gb3: Globotriaosylceramide
HR: Hazard ratio
ICD: Implantable cardioverter defibrillator
LVMI: Left ventricular mass index
LVMMI: Left ventricular myocardial mass index
Lyso-Gb3: Globotriaosylsphingosine
MSSI: Mainz severity score index
NA: Not applicable
NR: Not reported
OR: Odds ratio
ROC: Receiver operating characteristic
SD: Standard deviation
TLR: Targeted literature review
